# Cervical myelopathy mistaken for complex regional pain syndrome: A case report

**DOI:** 10.1097/MD.0000000000039173

**Published:** 2024-10-11

**Authors:** Jiwon Bak, Byeongmun Hwang

**Affiliations:** a Department of Anesthesiology and Pain Medicine, Kangwon National University Hospital, School of Medicine, Kangwon National University, Chuncheon, Republic of Korea.

**Keywords:** case report, cervical cord, complex regional pain syndrome, hyperalgesia, neuralgia, spinal cord compression

## Abstract

**Rationale:**

Degenerative cervical myelopathy (DCM) is characterized by spastic gait impairment, upper limb dysfunction, and sphincter disturbances. The pathological mechanism involves a combination of mechanical compression and ischemic processes, which are most commonly associated with the narrowing of the vertebral canal. However, DCM requires differential diagnosis from diseases of the central nervous system that cause neuropathic pain, such as complex regional pain syndrome (CRPS) and postherpetic neuralgia.

**Patient concerns:**

This report presents a case of DCM misdiagnosed as CRPS. Delayed diagnosis can lead to residual symptoms and functional disability.

**Diagnoses:**

Definitive diagnosis requires a correlation between physical findings and imaging results. Magnetic resonance imaging is the modality of choice, and spinal cord compression is the hallmark finding.

**Interventions:**

Anterior cervical discectomy and fusion.

**Outcomes:**

At the 8-week postoperative follow-up, the patient reported reduced pain. Arm function was almost normal, and although the gait was unstable, he was able to walk without assistance.

**Lessons:**

DCM can be easily confused with CRPS or postherpetic neuralgia. Therefore, physicians should consider the presence of different neuropathic pain syndromes when neuropathic pain develops. Patients with prior conditions affecting the cervical spine should be aware of the potential development of cervical myelopathy.

## 1. Introduction

Degenerative cervical myelopathy (DCM) is characterized by spastic gait impairment, upper limb dysfunction, and sphincter disturbances. The pathological mechanism involves a combination of mechanical compression and ischemic processes that are commonly associated with disc herniation or degenerative changes.^[1,2]^ Delays in diagnosis and treatment can lead to poor outcomes and lifelong disabilities.^[3]^

Complex regional pain syndrome (CRPS) is a life-threatening condition that usually affects the extremities after trauma or nerve injury.^[4,5]^ It commonly presents with allodynia, hyperalgesia, skin temperature changes, and edema. Patients with CRPS exhibit a constellation of symptoms throughout the disease course, including sensory abnormalities, autonomic signs, and motor dysfunction. The signs and symptoms of CRPS can be easily confused with those of DCM.

**Figure 1.**
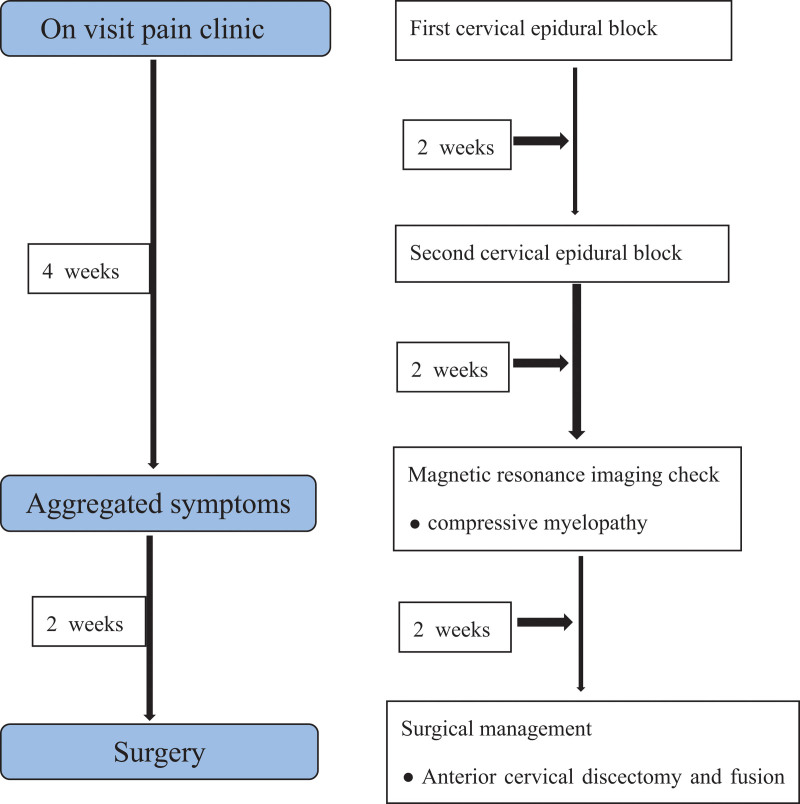
Timeline of the case with degenerative cervical myelopathy.

A DCM requires differential diagnosis from diseases of the central nervous system that cause neuropathic pain, such as CRPS and postherpetic neuralgia. Accurate diagnosis and appropriate treatment in the early stages of the disease are associated with a good prognosis and prevention of complications such as residual symptoms and functional disability.^[1–5]^ Therefore, physicians should consider the presence of different neuropathic pain syndromes during its development. In the present case, we immediately performed magnetic resonance imaging (MRI) when the patient did not respond to treatment and symptoms worsened. Subsequently, a joint consultation with a neurosurgeon was conducted on the basis of the patient’s MRI findings and symptoms. After consultation, surgery was performed to improve symptoms and prevent complications. After surgery, the patient experienced reduced pain and improved symptoms. Early diagnosis and active treatment are necessary to achieve optimal functional outcomes and reduce pain (Fig. 1). Here, we report a case of DCM misdiagnosed as CRPS.

## 2. Case report

A 52-year-old man (weight: 73 kg; height: 171 cm) was referred from the Department of Neurology to the pain clinic for neck and arm pain after a neurologist suspected the patient’s symptoms resulted from CRPS. In the Department of Neurology, the patient received medications including gabapentin, duloxetine, and tramadol for 2 weeks. Despite receiving medication, the patient persistently complained of pain in the neck and both arms. He also complained of paresthesia in the left arm, decreased range of motion in the right shoulder, and a tingling sensation in both arms.

The patient presented with pain in the neck and both arms that had begun 3 years previously. At that time, the patient was diagnosed with degenerative cervical spondylosis, herniated intervertebral discs, and loss of cervical curvature. He had a medical history of hypertension, and his right leg had been amputated 10 years prior in an industrial accident. At the pain clinic, the patient complained of severe radiating pain in both arms, with a visual analog scale score of 8. The pain in both arms worsened after heavy posterior extension of the neck in a massage chair 2 weeks prior to presentation. Physical examination revealed edema in the left and lower arms with no change in skin color. Neurological examination revealed neck stiffness, numbness, allodynia, and hyperalgesia in both upper limbs, all of which were more prominent on the left side. The patient presented with a distal weakness of 4/5 in the upper limbs and 5/5 motor power in the bilateral lower limbs. We diagnosed CRPS because his symptoms met the revised Budapest criteria.^[5]^ The patient fulfilled 3 of the 4 symptom criteria (allodynia, decreased range of motion, and asymmetry of edema).

**Figure 2.**
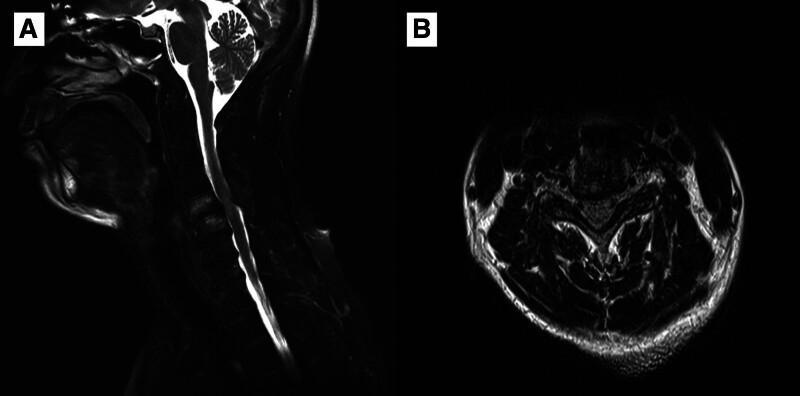
Magnetic resonance imaging in sagittal T2-weighted (A) and axial T2-weighted (B) sequences demonstrates central canal stenosis of C4-C5 due to disc herniation, ligamentum flavum thickening, and intramedullary T2 high signal intensity (evidence of myelopathy).

Owing to his unbearable pain, a cervical interlaminar epidural injection of 6 mL of lidocaine 0.5% and dexamethasone 5 mg of dexamethasone was administered under fluoroscopic guidance at the C6-7 level. The patient’s symptoms improved slightly, and the visual analog scale score decreased by 3 to 4 points 10 days after administration of the cervical epidural block. However, 10 days later, the symptoms were similar to those observed before the cervical epidural block. Two weeks after the administration of the first cervical epidural block, a second cervical epidural block was performed and the medication regimen was changed to 1800 mg/d gabapentin (twice the previously prescribed dose). When visiting the pain clinic 4 weeks after the first cervical epidural block, the patient complained of persistent pain and limb weakness that had worsened 3 days prior to presentation. He exhibited paresthesia in both arms, severe pain in the neck and upper extremities, and gait instability, and required walking assistance. Physical examination revealed signs of upper motor neurons, such as increased tone and brisk reflex in the upper limbs (3 + bilaterally in the biceps) and a positive Hoffmann sign. The patient denied any bowel or bladder disturbances. MRI of the cervical spine was performed to differentiate between conditions such as hematoma, infection, and myelopathy. The MRI showed left foraminal and central stenosis of C4-C5 with bulging discs, osteophytes, and facet arthrosis, causing compressive myelopathy (Fig. 2). Electromyography revealed a moderate motor pathway dysfunction from the cerebrum to both upper extremities. The modified Japanese Orthopedic Association score was 10 (3 + 3 + 1 + 3).^[1,6]^ After the diagnosis of DCM, the patient was referred to a neurosurgeon for a joint consultation.

**Figure 3.**
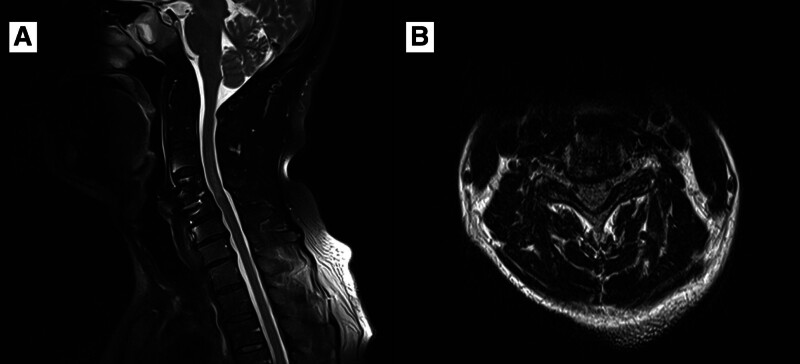
Postoperative magnetic resonance imaging in sagittal T2-weighted (A) and axial T2-weighted (B) sequences demonstrates C4-C5 anterior cervical discectomy and fusion without cord compression.

Surgical management was indicated and performed by a neurosurgeon 2 weeks after the symptoms worsened. Using an anterior approach, the spinal cord was decompressed at C4-C5 and fused to an interbody device. The patient’s surgical and postoperative course was uneventful. He was transferred to a rehabilitation facility, where gradual improvement in neurological deficits was observed. At the 8-week follow-up, the patient complained of mild pain in both arms and gait instability; however, he was able to walk without assistance. At that time, the medication was changed to pregabalin (150 mg/d). Function was almost normal, and there were no sphincter disturbances. A follow-up MRI of the cervical spine showed normal alignment without cord compression or old signal changes (Fig. 3).

## 3. Discussion

Spinal cord compression results in progressive neurological decline and affects patients’ quality of life. If left untreated, it can result in tetraplegia and wheelchair dependence. Conservative treatment of symptoms and regular follow-up to monitor progression may be offered to patients with mildly stable myelopathy. Conservative treatments include physical therapy, immobilization with hard or soft cervical collars, cervical traction, massage, spinal manipulation therapy, spinal injections, and the avoidance of high-risk activities.^[3]^ Although conservative treatment is an acceptable initial approach for patients with mild-to-moderate symptoms, 20% to 60% of these patients show deterioration of their condition over 3% to 6 years and may eventually require surgical intervention.^[3]^ DCM typically worsens over time. Delayed diagnosis and treatment can lead to poor outcomes and lifelong disability. Therefore, it is important to promptly recognize, evaluate, and appropriately refer these patients. Findings from 746 patients with myelopathy indicated that treatment within 6 months of symptom onset offers the best chance of recovery.^[7]^ In the present case, surgical treatment was offered within 2 months of the onset of neurological symptoms. Prompt diagnosis and appropriate intervention are key to managing cervical myelopathy. In the present case, early diagnosis and active treatment may have contributed to the good prognosis.

DCM is a common, nontraumatic, and progressive form of spinal cord compression.^[3]^ The average age at presentation is 64 years; it is more common in men, and C5-C6 is the most commonly affected level, followed by C6-C7 and C4-C5.^[3]^ In the present case, the patient was a 52-year-old man, and cord compression affected level C4-C5. Mechanical compression of the spinal cord is an underlying cause of DCM. Degeneration and deterioration of the intervertebral disc can lead to collapse, subsequent posterior bulging, and cord compression, resulting in cervical myelopathy. During flexion and extension of the cervical spine, the cervical cord is compressed by various pathological changes that exacerbate the signs and symptoms. In the present case, the patient’s signs and symptoms worsened after excessive neck movement. Common symptoms and physical examination findings include a decreased and possibly painful range of the cervical spine, neck pain or stiffness, paresthesia in the upper or lower extremities, lower extremity weakness, decreased hand dexterity, hyperreflexia, clonus, and bowel or bladder dysfunction.^[1,2]^ Deep tendon hyperreflexia is a key finding in myelopathy.^[3]^ Physical examination findings (e.g., Babinski, Hoffmann, and inverted supinator signs) associated with DCM tend to be more specific than sensitive. Loss of C5 (biceps) in the presence of a brisk C6 (brachioradialis) reflex is a pathognomonic physical examination finding of spinal cord compression at C4-C5. In the present case, the patient presented with upper motor neuron signs in the limbs, such as hyperreflexia in the biceps and positive Babinski and Hoffman signs.

A definitive diagnosis requires a correlation between physical and imaging findings. In the present case, myelopathy due to compression was suspected on physical examination and was confirmed by MRI. Physical examination revealed neck pain and stiffness, numbness, and allodynia in both arms, lower limb weakness, and deep tendon hyperreflexia. MRI is the imaging modality of choice for patients with suspected DCM, with the hallmark finding being squamous cell cord. In the present case, cervical MRI showed cord compression due to central canal stenosis of C4-C5 and disc herniation, ligamentum flavum thickening, and intramedullary T2 high signal intensity (Fig. 2); all findings were suggestive of myelopathy.

Symptoms of DCM can be persistent, fluctuating, or transient,^[3]^ and the evolution of symptoms is a consistent feature. Most patients present with symptoms that persist for months and then worsen; however, the progression rates vary. Symptoms may precede objective examination. These features of DCM are mild and difficult to observe during the initial disease stages. Nonspecific and subtle early features that overlap with other neurological conditions can delay diagnosis, and incomplete neurological assessments by professionals with poor awareness of the disease can also contribute to delays. A retrospective study of the medical records of 42 patients who underwent surgery for DCM noted an average delay of 2.2 years from symptom initiation to diagnosis.^[8]^ Forty-three percent of these patients had symptoms of numbness and pain in their hands and were initially diagnosed with carpal tunnel syndrome. Diseases of the central nervous system that cause neuropathic pain, such as CRPS and postherpetic neuralgia, require differential diagnosis from DCM based on physical examination. Initial symptoms of CRPS include severe burning pain, increased skin temperature, rapid nail and hair growth, and erythema. As the disease progresses, hair growth slows, the nails become more brittle, and muscle weakness begins. Eventually, irreversible muscle weakness, decreased muscle mass, and intractable pain can develop.^[4]^ This pain begins at the site of initial trauma and then spreads regionally without a specific dermatomal distribution. Cremer et al reported that CRPS is associated with myelopathy.^[8]^ The presentation of CRPS in patients with recent spinal cord injury can be easily confused with dysesthetic or radicular pain. Radicular pain following spinal cord injury occurs with a distinctive dermatomal or nerve root distribution in the injury zone. Dysesthetic pain can be easily confused with CRPS, because both diffuse burning pain and tactile defensiveness may be observed at or below the injury zone.^[4,5,9]^ Postherpetic neuralgia is often observed in patients with CRPS-like symptoms.^[10]^ Therefore, physicians should consider the presence of different neuropathic pain syndromes during its development.

Surgery is recommended for patients with moderate-to-severe or rapidly progressive disease. The modified Japanese Orthopedic Association score can be used to assess the severity of myelopathy based on presenting symptoms.^[1,6]^ The mJOA score in the present study was 10. This corresponds to a “severe” grade of DCM. Therefore, surgery was considered necessary. The overall goal of surgery is spinal cord decompression and stabilization to prevent neurological compromise. Generally, the anterior approach is preferred when 1 or 2 vertebral levels are involved, whereas the posterior approach is preferred when more than 2 vertebral levels are involved.^[11,12]^ In the present case, medication and a cervical epidural block had limited effects. Additionally, the patient exhibited sudden changes in symptoms (weakness in both arms and gait instability).^[13]^ Therefore, surgery was performed to improve the symptoms and prevent further neurological compromise. Surgical decompression can halt disease progression; however, the regenerative capacity of the spinal cord is limited and damage is often permanent. Therefore, it was not possible to predict the long-term surgical outcomes. Maximal recovery occurred at approximately 6 to 12 months of age.^[2]^

Residual symptoms are likely to be permanent and should be managed appropriately. Functional deficits are common and the most troublesome symptom is pain. Patients should be advised that complete resolution of pain is unlikely, although neuropathic analgesics and antispasticity medications can be used. Early referral to a specialized pain clinic is often helpful. Physicians should ask patients to report any worsening or new signs or symptoms because untreated regions of the cervical spine may further degenerate and cause spinal cord compression.

## 4. Conclusions

DCM in patients with cervical spinal stenosis and disc herniation can be easily confused with CRPS or radicular pain. Therefore, physicians should consider the presence of different neuropathic pain syndromes during its development. Patients with prior conditions affecting the cervical spine should be aware of the potential development of cervical myelopathy. If there is a change in symptoms in a patient with a preexisting disease of the cervical spine, early detection of the disease through detailed physical and imaging examinations would contribute to a good prognosis.

## Acknowledgments

The authors would like to thank Editage (www.editage.co.kr) for English language editing.

## Author contributions

**Conceptualization:** Jiwon Bak, Byeongmun Hwang.

**Data curation:** Jiwon Bak, Byeongmun Hwang.

**Investigation:** Jiwon Bak, Byeongmun Hwang.

**Writing—original draft:** Byeongmun Hwang.

**Writing—review & editing:** Byeongmun Hwang.
